# Genotype-Specific vs. Cross-Reactive Host Immunity against a Macroparasite

**DOI:** 10.1371/journal.pone.0078427

**Published:** 2013-10-22

**Authors:** Christian Rellstab, Anssi Karvonen, Katja-Riikka Louhi, Jukka Jokela

**Affiliations:** 1 Department of Biological and Environmental Science, University of Jyväskylä, Jyväskylä, Finland; 2 Biodiversity and Conservation Biology, Swiss Federal Research Institute WSL, Birmensdorf, Switzerland; 3 Aquatic Ecology, Eawag (Swiss Federal Institute of Aquatic Science and Technology), Dübendorf, Switzerland; 4 Institute of Integrative Biology, ETH Zürich, Zürich, Switzerland; Institut Pasteur, France

## Abstract

Vertebrate hosts often defend themselves against several co-infecting parasite genotypes simultaneously. This has important implications for the ecological dynamics and the evolution of host defence systems and parasite strategies. For example, it can drive the specificity of the adaptive immune system towards high genotype-specificity or cross-reactivity against several parasite genotypes depending on the sequence and probability of re-infections. However, to date, there is very little evidence on these interactions outside mammalian disease literature. In this study we asked whether genotype-specific or cross-reactive responses dominate in the adaptive immune system of a fish host towards a common macroparasite. In other words, we investigated if the infection success of a parasite genotype is influenced by the immunization genotype. We reciprocally immunized and re-exposed rainbow trout (*Oncorhynchus mykiss*) to a range of genotypes of the trematode eye fluke *Diplostomum pseudospathaceum*, and measured infection success of the parasite. We found that the infection success of the parasite genotypes in the re-exposure did not depend on the immunization genotype. While immunization reduced average infection success by 31%, the reduction was not larger against the initial immunization genotype. Our results suggest significant cross-reactivity, which may be advantageous for the host in genetically diverse re-exposures and have significant evolutionary implications for parasite strategies. Overall, our study is among the first to demonstrate cross-reactivity of adaptive immunity against genetically diverse macroparasites with complex life cycles.

## Introduction

In nature, hosts are typically exposed to and infected with multiple parasite genotypes [1,2]. These infections are ecologically dynamic, depending, for example, on the variation in host ecology, spatial distribution of parasite intermediate hosts, and temporally fluctuating risk of exposure driven by seasonality of the release of parasite infective stages (e.g., [3-6]). This has important implications for host-parasite co-evolution in terms of selection pressures that shape host defence systems and parasite strategies to elude those defences [7]. The immune system of a vertebrate host has two principal ways of fighting parasitic infections [8]. The innate immune system is activated rapidly, targeting pathogens that invade the host for the first time. The adaptive (acquired) immune system, on the other hand, is activated more slowly, but features a highly specific and long-lasting immunity against secondary infections. In long-lived vertebrate hosts, adaptive immune processes play an important role as the hosts spend only a small fraction of their lives unexposed and unimmunized. 

As immunological experience of infections accumulates with time, specific and non-specific responses shape subsequent infections and parasite establishment to an increasing degree [9]. Two types of scenarios can be considered. First, host responses might be specific against individual parasite genotypes (or strains) and provide protection only if the host is re-exposed to the same genotype. Such responses have been described, for example, in mammals infected with microparasites like viruses, bacteria and flagellates (e.g., [10-12]). Alternatively, specific responses evoked against one genotype (typically the first one infecting a host) may be cross-reactive and provide at least a partial protection against other genotypes of the same species (e.g., [13]), or even those of different species (e.g., [14]). This mechanism underlies the long-lasting effect of vaccination against, for example, influenza (e.g., [15]). To date, most studies testing different scenarios for specificity of adaptive immunity focused on infectious diseases or microparasites, while much of the empirical work on evolutionary ecology of specificity of host defences against macroparasites has been based on innate immune defence (e.g., [16]). Specific responses against macroparasites are important, however, as the diversity of such infections, like that of microparasites, is typically high (e.g., [17]). Macroparasites show also very different modes of establishment, host exploitation, growth and reproduction compared to microparasites [18], resulting in diverse fitness consequences and selection towards different defensive scenarios in the hosts. 

The two scenarios of defence described above have important implications for both host and parasite. Assuming that immune defence is costly and the costs increase with the diversity of specific responses needed for an effective defence [19-21], a genotype-specific memory would only be advantageous if there is a high chance of re-exposure from a previously encountered parasite genotype. This is possible, for example, if hosts stay within a narrow home range and parasite prevalence (i.e., genotype diversity) is low in intermediate hosts that transmit the parasites. In contrast, if re-exposure comes mostly from different genotypes, selection should favour cross‑reactivity as it presents a more widely applicable defence [19]. From the parasite’s perspective, cross-reactive host responses could be beneficial if they reduce competition among individual parasites for limited host resources (i.e., competitive exclusion via host immunity, [9]). However, this could also impose strong divergent selection on parasites to escape the host's cross-resistance and maintain higher infection rate and host exploitation also in immunized hosts. Genotype-specific immune responses, on the other hand, could lead to higher reproductive success of the parasites in terms of outbreeding as it increases the likelihood of a multiple-genotype infection in an intermediate host, followed by the transmission of a genetically diverse community to the definitive host [17]. However, very few studies have tackled the associations between different types of host responses although this represents the necessary first step to address these different scenarios.

In this study, we experimentally tested between genotype-specific and cross-reactive host immune responses in a fish-trematode system, rainbow trout (*Oncorhynchus mykiss*) infected with the eye fluke *Diplostomum pseudospathaceum*. In this well-studied system, several wild fish species, including rainbow trout, are commonly infected with high numbers of parasites [22-24]. For example, in the study of Wootten [24], 95.8% of the rainbow trout from an English reservoir carried in average 47.8 and up to 552 lens parasites. Fish acquire partial immunity within a few weeks after the first exposure, meaning that the immune system significantly reduces the number, but not necessarily the prevalence, of parasites establishing in subsequent exposures [25-28]. However, it is unknown if the adaptive immune responses are genotype-specific or cross-reactive although genotype-specific responses of the innate immune system have been described [16]. In this system, parasite infective stages (clonal cercarial genotypes) are released from the first intermediate hosts (snail) in very high numbers over several weeks to infect the second intermediate (fish) host [29]. Therefore, host re-exposure from the same parasite genotype is certainly possible. It is also typical that fish carry a high number of different parasite genotypes [17], suggesting genetically diverse re-exposure over time. 

We addressed the hypotheses of genotype-specificity and cross-reactivity of the host immune system by conducting a factorial infection experiment, where fish immunized with single parasite genotypes were subsequently re-exposed to either the same or different genotypes. Beside high parasite genotype-specific variation in infection success in naïve hosts, we found dominance of cross-reactive host responses in the re-infection, suggesting that apparent competition among parasite genotypes is strong. 

## Material and Methods

### Study organism

The life cycle of *D. pseudospathaceum* includes three hosts [25]. Adult specimens reproduce sexually in the intestine of fish eating birds. Parasite eggs are released with bird faeces into water, where they develop to miracidia and infect their first intermediate host, a freshwater snail. In the snail, parasites reproduce asexually and leave the snail as thousands of genetically identical cercariae during a period of several weeks [29]. Cercariae infect the second intermediate hosts, a freshwater fish, by penetrating the skin and gills, and migrating to the eye lenses. The lens itself is an immunologically privileged site as it lacks blood circulation, but the parasites are exposed to the fish immune system for a maximum of 24 hours while migrating towards the eye [25]. In the lens, parasites develop to metacercariae and can cause significant fitness consequences for the fish. For example, heavy infections in the eye lead to impaired growth [30] and increased susceptibility to predation [31]. The life cycle is completed when an infected fish is eaten by a bird. In the final host, the parasite is also capable of selfing [25], but population genetic analyses [32] do not support that this happens often.

### Collection of parasites

*Lymnaea stagnalis* snails were collected from the shallow littoral zone of Lake Konnevesi (Finland, 62° 37' N, 26° 21’ E) at the end of June 2010. A sampling permission was not required, because the sampling location is not a nature reserve and *L. stagnalis* is not protected or endangered in Finland. Individual snails were placed in small containers with lake water (20°C) and checked 2 hours later for the production of *D. pseudospathaceum* cercariae. Previous work had shown that *L. stagnalis*, a common host to a range of trematode taxa, is infected with only one species of *Diplostomum*, *D. pseudospathaceum* [22,32]. However, snails can be infected with multiple genotypes of *D. pseudospathaceum* [33]. Since we needed single genotype infected snails in the experiments, 16 cercariae were randomly picked and frozen from each infected snail. DNA from individual cercariae was extracted with Chelex 100 resin [34] and the number of multi-locus parasite genotypes per snail was determined using three highly polymorphic microsatellite markers (Diplo06, Diplo09, Diplo23) designed for *D. pseudospathaceum* [32,35]. As two different genotypes can exhibit the same multi-locus genotype and hence double-infected snails might be misinterpreted as single-infected hosts, we calculated the unbiased probability index (PI) and the random matching probability (MP) using Gimlet 1.3.3 [36] and a larger set of 21 different genotypes from the same lake and year. The PI ranged from 10^-4^ to 10^-7^ depending on the locus, and the MP from 10^-4^ to 10^-8^ depending on the genotype, indicating that the likelihood for such a misinterpretation is extremely low.

### Immunization of fish

Juvenile rainbow trout (*O. mykiss*, length: 7.1 cm ± 0.2 SE) were obtained from a groundwater-fed fish farm in Central Finland in the beginning of July 2010, ensuring that fish had no prior exposure to the parasite because of absence of snails in the groundwater. Fish were divided into nine groups, each consisting of 130 individuals, and the groups were randomly placed in nine fish tanks containing 200 l of aerated groundwater (15.8°C ± 0.0 SE). Seven of the fish groups received an immunization treatment (exposure to a low-level natural infection), each with a different parasite genotype, while two tanks were kept as uninfected controls. Parasite genotypes were retrieved from seven single genotype infected snails (S2, S7, S10, S11, S13, S14, and S16) that were kept in small containers at 18°C for the production of cercariae for four hours. Cercarial densities in the containers were determined by taking five 1 ml samples from each container. During the exposure of the fish groups, the water level in the tanks was reduced and the water supply stopped for 30 minutes. Each fish group was exposed to an estimated total number of 1300 cercariae (10 cercariae per fish). After the exposure, water supply was switched on and the water level was brought back to normal. Infected snails were subsequently stored at 4°C and fed *ad libitum* with lettuce for five weeks. Once a week, snails were brought to the laboratory for some hours (20°C) to stimulate cercarial release. One snail (S16) died during this period.

Fish were fed with commercial fish pellets (approx. 3% of the average fish weight per day) and kept in the tanks for five weeks (16.3°C ± 0.1 SE), which is enough for the development of adaptive immune responses (e.g., [27,28,37]). Once a week, fish groups were moved randomly between the tanks to exclude tank specific effects for the development of immunity, and the tanks were cleaned and emptied. As the cercariae have a maximum lifespan of 20-36 h outside the host [38] and the first fish group movement was done after 4 days, infection by other parasite genotypes could be excluded. Average water temperature did not differ among the groups (ANOVA, random factor, nested in immunization treatment, F_7,288_=0.216, p=0.981) or between the treatments (fixed factor, naïve or immunized, F_1,7_=0.261, p=0.625). After five weeks, cumulative mortality was between 0 and 2.3% depending on the fish group. The number of dead fish did not differ between immunized and control groups (t-test, n=2 and n=7, resp., p=0.416). Average length of the fish after the immunization period was 9.6 cm ± 0.1 SE.

### Re-exposure of fish

Six snails survived the five-week immunization period and were used in re-exposing the fish. To compensate for the one dead snail used in the immunization trial and to increase the power of the statistical analyses, we included two new single-genotype infected snails (S4 and S6) to the re-exposure design. These snails had been sampled at the same time and treated exactly the same as the original snails, but had not been used in immunizing the fish. All snails were taken out of the cold room and allowed to release cercariae in a small amount of water (17°C) for four hours. The total number of cercariae in the suspension from each snail was estimated by taking five 1 ml subsamples. The eight fish groups (the seven immunized groups and the uninfected control fish pooled from the two tanks) were then exposed reciprocally to the eight parasite genotypes. Each exposure combination included ten fish, totalling 640 fish. Fish were placed individually in 5 dl of water (17°C) and exposed to an estimated dose of 50 cercariae. After 30 min, each treatment group of ten fish was placed in a 35 x 35 x 35 cm mesh cage and the cages were placed randomly in five holding tanks (1500 l) with continuous water flow (17°C). Fish were maintained in these conditions for 72 h to allow parasite establishment after which they were euthanized with an overdose of MS‑222 anaesthetic. Fish were measured for length and dissected for the number of parasites in the eye lenses. “Old” parasites originating from the immunization were separated from those originating from the re-exposure ("new infections") according to their size and morphology; e.g., newly established parasites are substantially smaller than parasites that are more than 5 weeks old [39]. Two fish escaped from their mesh cages during parasite establishment and were excluded from the analysis. Moreover, six fish died before dissection and their parasite number could not be determined. All experiments were carried out with permission (license number ESLH-2008-05938/Ym-23) from the National Animal Experiment Board (ELLA) in Helsinki, and complied with the animal care legislation of Finland.

### Statistical analyses

We performed two analyses of covariance (ANCOVA) using log-transformed (ln+2) number of parasites in each fish (only new infections) as a dependent variable. In the first analysis (ANCOVA 1), we tested for a general effect of immunization, i.e., whether naïve control fish had higher parasite numbers after the re-exposure compared to immunized fish. Immunization treatment (immunized or naïve) was used as a fixed factor and parasite genotype (re-exposure genotype) as a random factor. Length was used as a covariate. In the second analysis (ANCOVA 2), we tested for a genotype-specific immunization effect. Consequently, data of control fish were excluded. Immunization genotype and re-exposure genotype were defined as a random factors and length was used as a covariate. A significant interaction between immunization genotype and re-exposure genotype would reveal genotype-specific responses of the adaptive immune system. Finally, we performed a custom hypothesis test using a contrast analysis to test how parasite genotypes perform in a re-exposure with the same genotype compared to a re-exposure with a different genotype. For the contrast analysis, only the six parasite genotypes that were used in both exposures were included.

A preliminary ANCOVA showed that fish length at the time of re-exposure was not randomly distributed among the fish groups, but depended on the immunization genotype (random factor, F_6,539_=4.186, p<0.001), the number of old infections (covariate, F_1,539_=35.874, p<0.001) and their interaction (F_6,539_=2.651, p<0.001). In other words, a higher number of old infections was found in larger fish of all fish groups, and this relationship was significant (α=0.05, tested using Pearson's correlation) in four of the seven fish groups. Two different processes might explain this pattern. Either the more infected fish grew faster during the establishment of immunity, or the larger fish in each tank received more parasites during the immunization procedure. Although the latter scenario is more likely, it is still in contrast to the usual pattern in this system where larger fish tend to receive fewer parasites (see the result of the re-exposure in this study). However, this deviating pattern may simply reflect the outcome of different experimental conditions. For example, in contrast to the re-exposure, fish were not individually exposed to the parasite and had more space to move in the tank during the immunization. Although a non-evenly distributed covariate is not optimal for randomization, we used it as a standard covariate because the overlap in length distribution of the fish groups was still considerable, and the length of fish (average difference in length was max. 6 mm among the fish groups) had a negligible impact on infection success in the re-exposure (estimated -0.1 parasites per mm in length). 

## Results

The average number of parasites resulting from the immunization experiment (old infections) in the immunized fish ranged between 6.3 and 11.6 parasites depending on the fish group. In one case (S7) the mean number of parasites per fish exceeded 10, suggesting an error in the exposure dose in this specific case. Among fish immunized with the same parasite genotype, the maximum standard error of number of old infections was 0.5. Only two fish did not become infected during the immunization procedure, resulting in an overall prevalence of 99.6%. All control fish remained uninfected during the five week period between immunization and re-exposure. 

**Figure 1:**
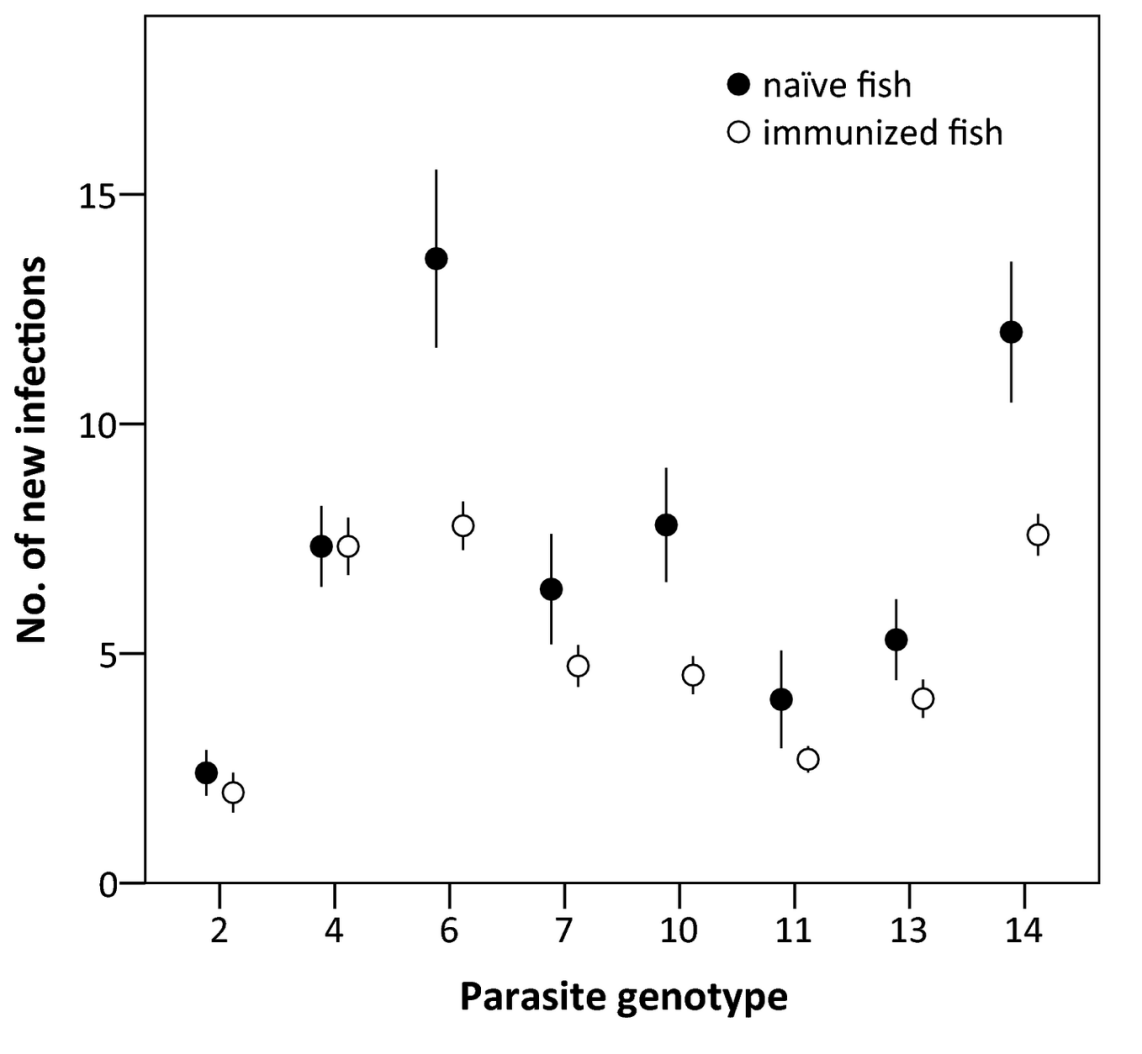
Infection success of *Diplostomum pseudospathaceum* genotypes in previously unexposed (naïve) and immunized juvenile rainbow trout. Error bars represent standard error.

**Figure 2:**
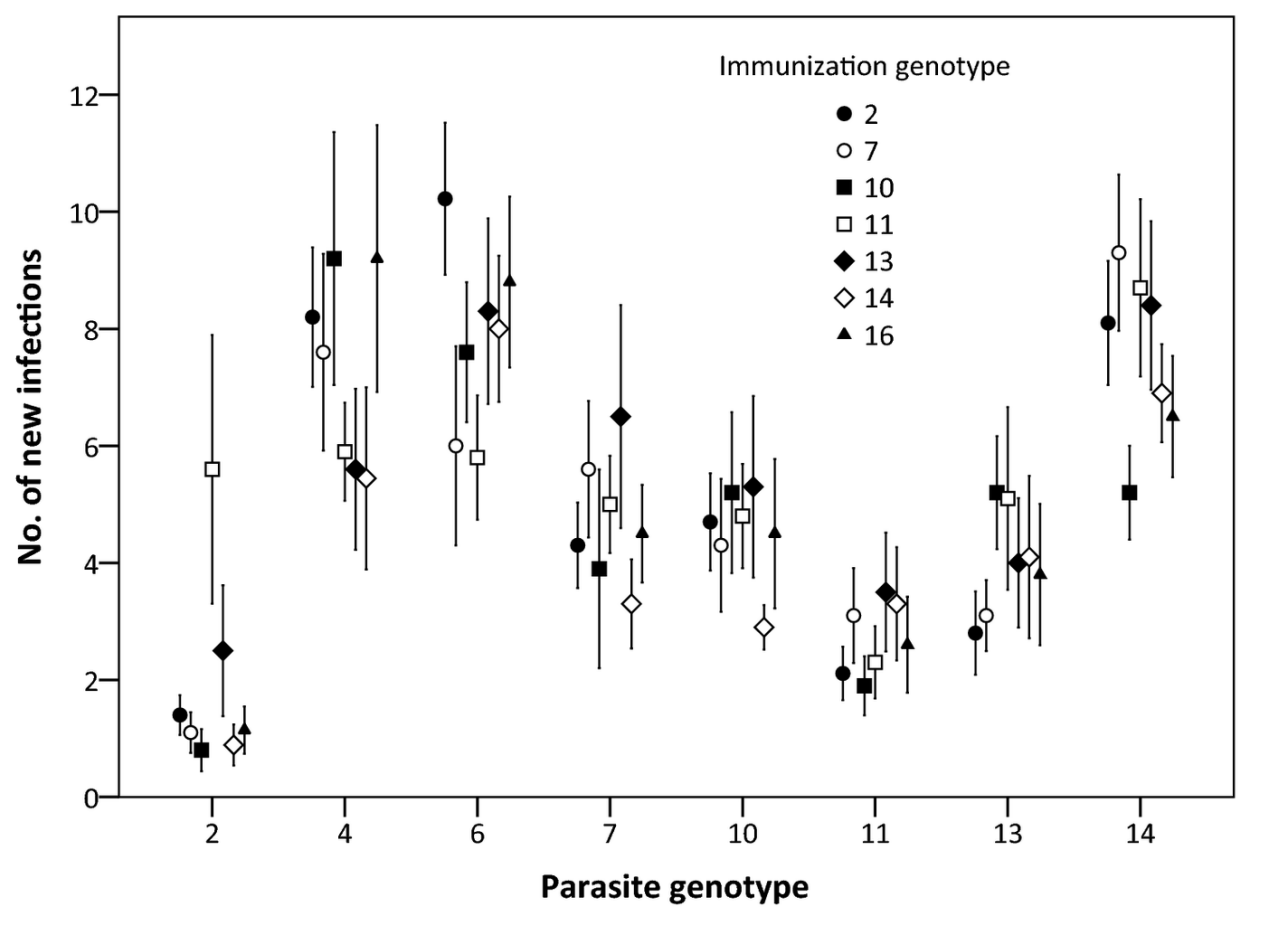
Infection success of *Diplostomum pseudospathaceum* genotypes in juvenile rainbow trout immunized with different parasite genotypes. The different immunization genotypes are given at the x-axis, the different re-exposure genotypes are marked with different symbols. Error bars represent standard error.

After the re-exposure, 97.5% of the naïve and 92.2% of the immunized fish harboured new infections (prevalence of infection). Average parasite infection success (only new infections) was 5-27% in naïve, and 4-16% in immunized fish groups, depending on the parasite genotype. Immunized fish acquired significantly fewer parasites in the re-exposure compared to control fish (ANCOVA 1, Table 1). Overall, the infection success was 31% lower in immunized fish (10.2 vs. 14.7%), but there were significant differences among the parasite genotypes. Only parasite genotype S4 did not have higher average infection success in naïve fish than in immunized fish (Figure 1). Moreover, the covariate length had a significant effect on infection success, with smaller fish receiving more parasites. In ANCOVA 2, the interaction between immunization genotype and re-exposure genotype was not statistically significant (Figure 2 and Table 2), indicating that there was no genotype-specific response. The significant effect of re-exposure genotype and length was confirmed in this analysis. Finally, infection success did not differ between fish that were re-exposed to the same parasite genotype or to a new one (custom hypothesis test, F_1,321_=0.051, p=0.822), also indicating cross-reactivity of the fish immune system.

**Table 1:** Result of the first ANCOVA testing the general effect of immunization.

Source	Type III Sum of Squares	df	MS	F	p
Intercept	100.806	1	100.806	278.856	<0.001
Length	20.189	1	20.189	93.057	<0.001
Immunization treatment (IT)	6.015	1	6.015	49.522	<0.001
Re-exposure genotype (RG)	34.464	7	4.923	40.453	<0.001
IT × RG	0.851	7	0.122	0.560	0.788
Error	133.423	615	0.217		

Effect of immunization treatment (immunized or previously unexposed fish) on the infection success (number of new infections at re-exposure) of eight different *Diplostomum* genotypes (re-exposure genotype, random factor) in juvenile rainbow trout. Length was used as a covariate.

**Table 2:** Result of the second ANCOVA testing the effect of immunization genotype and re-exposure genotype.

Source	Type III Sum of Squares	df	MS	F	p
Intercept	76.517	1	76.517	227.271	<0.001
Length	16.038	1	16.038	72.166	<0.001
Immunization genotype (IG)	1.829	6	0.305	1.105	0.375
Re-exposure genotype (RG)	62.667	7	8.952	32.437	<0.001
IG × RG	11.598	42	0.276	1.242	0.148
Error	110.232	496	0.222		

Effect of immunization genotype and re-exposure genotype (both random factors) on the infection success of *Diplostomum* (number of new infections at re-exposure) in juvenile rainbow trout. Length was used as a covariate.

## Discussion

Long-lived vertebrate hosts may encounter the same clonal parasite strains or genotypes repeatedly during their life depending on the ecological dynamics of the hosts and parasites. This should favour the evolution of the immune system towards specific immune responses against secondary exposures (adaptive immunity including an immune memory). There is evidence to support this, for example, in infectious diseases and microparasites (e.g., [10-12]). However, at the same time, selection pressures on hosts in terms of costs associated with highly specific immune responses [19-21], and those on parasites in terms of competitive exclusion of secondary infections, may favour broad-scale cross-reactive host responses. Overall, these different scenarios are at the core of evolutionary ecology of parasite-parasite and host-parasite interactions. For example, the degree of specificity of immune responses is important in vaccine development (e.g., [15]), in immune-mediated apparent competition between parasite strains [9], and also as a prerequisite for host-parasite co-evolutionary hypotheses like the Red Queen dynamics [40-42]. However, this has received very little attention in vertebrate host-macroparasite systems (but see, e.g., 43-46). In the present study, we contrasted genotype-specific and cross-reactive host responses in fish (*O. mykiss*) and found evidence for cross-reactivity against genotypes of a complex life cycle trematode (*D. pseudospathaceum*).

Initial low-dose exposure of fish to *Diplostomum* genotypes resulted in lower infection success upon secondary encounter compared to fish that had never encountered the parasite. This indicates that the fish became successfully immunized. The contact with *Diplostomum* antigens leads to a detectable activation of the adaptive immune system in rainbow trout that results in partial immunity upon following exposures (reviewed in 25). The response involves several interacting factors and immunological pathways like interleukins, MHC, T- and B-cells (for a review of fish immune responses see 47). Therefore, we did not measure any specific immunological parameters in this study, but used the total parasite number as a response variable that included all immunological processes above, an approach commonly adopted in evolutionary ecology [19]. The effect of immunization (31% average reduction in infection success) was relatively low, but it is well in accordance with the broad range of immune efficacies reported in this system [20-90%, 25,26-28,48]. Such a variation in the effect of immunization suggests that experimental immunization and re-exposure conditions (e.g. temperature, parasite dose, age, and species of fish, etc.) are likely to play an important role in determining the efficacy of the adaptive immunity. 

Most importantly, although we detected parasite genotype-specific infectivity, we did not find evidence for genotype-specific immune responses; the interaction between immunization genotype and infection genotype was not significant, and the re-exposure of fish to the same genotype did not result in lower parasite numbers compared to re-exposure to a different genotype. These results suggest that immunization with one parasite genotype resulted in cross-immunity against the other genotypes. While our results are in accordance with findings from non-reciprocal experiments with nematode clones [43-45], they are in contrast to a more recent study [46] in a schistosome-mouse system. Beltran et al. [46] found that the success of the re-infecting parasite genotype was dependent on the identity of the immunization genotype, as parasites that were genetically more similar had lower infectivity at re-exposure. This suggests high system-specific variation in these processes which is likely to result from differences in the evolutionary ecology of these host-parasite interactions. 

In evolutionary terms, competing parasite genotypes should evolve towards differentiation in antigen structures to escape cross-reactive immune responses of the host [18]. This would seem particularly beneficial in complex life-cycle parasites like *Diplostomum* as a genetically heterogeneous community in an intermediate host increases the likelihood of outcrossing among different clones in the definitive host [17]. Moreover, genetically heterogeneous attack has recently been shown to increase the infection success of the parasites in association with host innate immune system [49]. The present result, however, does not support these evolutionary scenarios. In contrast, cross-reactivity in host responses following an exposure to a single parasite genotype should result in decreased heterogeneity in genotype composition infecting a fish. However, in our previous study we did not find any indication of inbreeding in *D. pseudospathaceum* [32], which might be due to the time lag between the first infection and establishment of immunity in the fish, the fact that immunity is only partial, or because the final bird hosts most likely acquire parasites from several fish individuals over a long period of time. Cross‑reactivity in fish might even be beneficial for the parasite if it prevents later-arriving genotypes from entering the lens and reduces the competition among the genotypes in the host eye [9]. Details of such interactions, however, are unknown.

It is also possible that the optimal strategy for host defence depends on the interactions between innate and adaptive branches of the immune system, as well as on specific details of the infection process. For example, compared to higher vertebrates, the adaptive immune system of fish is relatively slow [47,50], and also shows lower antibody diversity [51]. Moreover, *Diplostomum* parasites are only exposed to the immune system for a short time (max. 24 hours, [25]) during cercarial migration in host body towards the eye. Under such circumstances, selection may not favour high investment of resources into the adaptive immune system with a specific memory, but rather lead to a fast and efficient reaction of the innate immune system [48]. Strong responses of the innate immune system against *Diplostomum* have been reported, for example, in sticklebacks [48], and these responses are also known to reduce the activation of the adaptive immune system [52]. 

The probability of re-exposure by the same genotypes, strongly driven by both parasite and host ecology, may also influence evolution of defence strategies. For example, if the fish hosts are exposed to a high number of random parasite genotypes (see 32 for the lack of population genetic structure in these parasites), there would be little selective pressure for the host to develop a genotype-specific immune response. This is also emphasised by the high inter-annual variability and turnover of the parasite population in a lake; genetically distinct parasite genotypes are continuously lost from the population through mortality of the infected snails and replaced by new sexually produced genotypes transmitted to snails from bird definitive hosts. However, in this system [29], as in other trematode-fish systems [53], the duration of cercarial production from individual infected snails typically exceeds the time needed for establishment of immunity, allowing re-exposure of one host individual to the same parasite genotype. Thus, different selective pressures for host defences may act depending on the diversity of parasite genotypes (i.e., overall infection prevalence in the snail population), specific habitat characteristics (e.g., size of a lake) and host ecology (e.g., habitat specificity of the fish).

Interestingly, recent studies in this system suggest rapid genotype-specific responses of the fish innate immune system without a previous encounter with the parasite [16], which is in accordance with the evidence from invertebrate studies showing previously unknown specificity of the innate immune system [54,55]. Our results also support this view if we look at how much the average infectivity of parasite genotypes varied when exposed to naïve host with the same genetic background (Figure 1). Combined with the present results, this represents an interesting pattern that contradicts the classical view of how innate and adaptive immune systems work. In this scenario, responses of the fast innate immune system would be genotype-specific, while the slow (until the establishment of immunity) adaptive immune system would be unspecific. However, it should be pointed out that Rauch et al. [16] used a fundamentally different approach to show the specificity of the innate immune system (testing host family × parasite genotype interactions), as well as different hosts species (sticklebacks), making these two studies difficult to compare. More research is needed to test if our findings represent general patterns or if they are fish species-specific. For example, *O. mykiss* is not a native species in Finland and has only a short co-evolutionary history with this particular *Diplostomum* species, which might contribute to the absence of genotype-specific responses. However, the fact that *O. mykiss* can establish partial immunity against a mixture [26-28] or single parasite genotypes (this study), suggests that genotype-specific responses are generally possible. Moreover, because the innate immune system of fish shows some degree of specificity [16] and we measured the reduction in number of established parasites that included the operation of the entire immune system, it is possible that these effects are not induced solely by the adaptive immune system.

Our result could also imply that hosts have difficulties in recognizing the specific antigenic variation among the parasite genotypes, resulting in inability of the host defence system to distinguish among them. This type of general defence response would be expected in systems where parasite-host local adaptation is hindered by high gene-flow and non-self recognition is based on non-specific features. Therefore, future studies should look also into immunological parameters to reveal if the detailed responses evoked by different parasite clones are similar. Generality of the results would also require comparisons of neutral/adaptive genetic variation and antigenic diversity within and among parasite populations.
